# Observational Research on Alcohol Use and Chronic Disease Outcome: New Approaches to Counter Biases

**DOI:** 10.1155/2013/860915

**Published:** 2013-07-09

**Authors:** Wenbin Liang, Tanya Chikritzhs

**Affiliations:** National Drug Research Institute, Curtin University, GPO Box U1987, Perth, WA 6845, Australia

## Abstract

*Background*. The frequently reported protective effects of moderate alcohol consumption in observational studies may be due to unadjusted bias. *Aim*. To examine two new approaches that account for unknown confounding factors and allow the application of intention-to-treat analysis. *Method*. This study used data from the 2008, 2009, and 2010 National Health Interview Surveys conducted in the United States. Unknown confounding effects were estimated through the association between parental alcohol use and health outcomes for children, because the presence of hypothetical physiological effects of alcohol can be ruled out for this association. In order to apply intention-to-treat analysis, previous alcohol use of former drinkers was obtained by using multiple imputations. Estimates with new adjustment approaches were compared with the traditional approach. *Results*. The traditional analytical approach; appears to be consistent with findings from previous observational studies; when two further adjustment approaches were used, the “protective” effects of moderate drinking almost disappeared. *Conclusion*. Use of a proxy outcome to estimate and control residual confounding effects of alcohol use and application of the intention-to-treat principle could provide a more realistic estimation of the true effects of alcohol use on health outcomes in observational epidemiological studies.

## 1. Introduction

There has been an ongoing debate as to whether moderate alcohol consumption imparts actual physiologically protective effects which measurably benefit human health or, alternatively, whether the observed associations may be due, at least in part, to methodological bias [1–10]. One of the systematic biases described by Fillmore et al. is that of misclassification error, where in many cohort studies, former drinkers are often mixed with lifetime abstainers who have never consumed alcohol and/or long-term abstainers [3, 11]. In addition, observed protective associations could be due to residual confounding effects produced by clusters of (both known and unknown) factors that strongly correlate with moderate drinking [1–10, 12]. 

Many confounding factors for patterns of alcohol use are clustered within the family such as socioeconomic determinants, environmental factors, lifestyle, and genetic susceptibility [13]. Previous research has demonstrated that better health status is more likely to be observed among children aged 17 years or younger whose fathers or mothers were current drinkers than those whose fathers or mothers were abstainers [12]. It is possible to obtain an estimate of family-clustered confounding effects for alcohol use by measuring the association between health outcomes for children and parental alcohol use, since confounding effects will remain even when exposure is absent (i.e., among children) [14].

In addition, in most observational studies, participants who used to drink alcohol but stopped sometime before the beginning of a study are often coded as “former drinkers” and separated from “current drinkers” in the analysis. However, the intention-to-treat analysis principle applied in clinical trials in order to avoid bias associated with exposed subjects who withdraw from treatment indicates that former drinkers should in fact be added back to a drinking category based on their previous alcohol consumption pattern [15]. Among studies where level of previous alcohol consumption among former drinkers may be unknown (e.g., cohort studies with participants in their mid-40s at baseline), a plausible estimate of previous alcohol use might be obtained by using multiple imputations, a common strategy to handle missing value [16]. In this paper we aimed to use these two new approaches to adjust for bias that may influence apparent associations between alcohol use and health status. 

## 2. Method

This study used data from the 2008, 2009, and 2010 National Health Interview Surveys (NHIS). Data from the three waves of surveys were combined and analyzed together. 

Details of the survey sampling strategy and data collection methods have been described elsewhere [17–19]. Briefly, the NHIS were nationally focused and conducted by the National Center for Health Statistics (NCHS), Centers for Disease Control and Prevention (CDC). All the surveys used the same sample design as the 2006 survey. The NHIS were conducted to provide comprehensive estimates of health indictors at the national level, and state stratified samples were drawn from all 50 states and the District of Columbia to ensure the samples are representative [17–19]. The NHIS collected basic demographics and information on health status from each household member. In addition, one randomly selected adult (>18 years), the key participant, was interviewed in detail regarding their health and health-related behaviour, including alcohol use in the last 12 months. Alcohol consumption levels (drinking patterns) were defined in the same way as the NHIS surveys [17–19] and grouped as follows: (1) lifetime abstainer, <12 drinks in lifetime; (2) former infrequent, 12+ drinks in lifetime but never as many as 12 in one year and none in the past 12 months; (3) former regular, 12+ drinks in lifetime, 12+ drinks per year, but none in past 12 months; (4) current infrequent drinker, 12+ drinks in lifetime and 1–11 drinks in past 12 months; (5) current light, 12+ drinks in lifetime, and no more than 3 drinks per week in past 12 months; (6) current moderate, 12+ drinks in lifetime, and 4–14 drinks per week (male) or 4–7 drinks per week (female) in the last 12 months; (7) current heavier: 12+ drinks in lifetime, and more than 14 drinks per week (male) or more than 7 drinks per week (female) in past 12 months; and (8) drinking status not reported. The outcome of interest was the health status of adults. The association between parental alcohol use and children's health status was used as a proxy measure for residual confounding effects. Adult health status was divided into two groups for comparison: excellent, very good, and good (adult good); and fair, and poor (adult low). Child health status was also divided into two groups: excellent, very good (child high); and good, fair, and poor (child low). About 15% of adult participants had fair or poor health status (adult low), and some 20% of children participants had good, fair, or poor health status (child low).

### 2.1. Data Analysis

In this study, the association between alcohol use and health status in adults was first estimated using the traditional approach. Poisson regression was used to predict the relative risk of undesirable health status (adult low) by alcohol use level, and models included the following control variables: gender, age strata, race, marital status, employment status in the last week, whether had private health insurance, achieved highest level of education, home tenure status, family income, number of family members under 18 years of age, and number of family members aged 65 or older. About 6% of participants had missing data in control variables and were therefore excluded from further analysis. To account for residual confounding effects, natural logarithms of the adjusted relative risk of undesirable health status among children by parental alcohol consumption level were added as an offset variable into the model. The adjusted relative risk of undesirable health status among children (child low) by the drinking status of their parents was estimated using Poisson regression while controlling all variables in standard approach (as above) plus gender and child age strata. 

To enable the use of intention-to-treat analysis, former drinkers (15%) and a small number of participants with unknown drinking status (2%) were treated as drinkers whose alcohol consumption level was unknown. Multiple multinomial logistic regression was then used to impute/predict their alcohol consumption levels based on the observed association between alcohol use and the socioeconomic demographics variables controlled in the standard approach among current drinkers. Fifty imputation estimations were obtained. Former drinkers and those with unknown drinking status were then added back into subgroups of drinkers based on imputation estimations. To perform intention-to-treat analysis, Poisson regression was rerun with abstainers and four levels of drinkers while controlling all variables included in the standard approach. A further analysis was performed to take both residual confounding effects and intension to treat into account by applying both the offset variable and intention-to-treat analysis within the Poisson regression model.

## 3. Results

In the traditional approach it was observed that compared to lifelong abstainers, moderate and light drinkers were significantly less likely to report a fair or poor health status, whereas former drinkers were significantly more likely to report a fair or poor health status. Similar “protective” effects were also observed for children when grouped according to their parental alcohol use (Table 2). The natural logarithms of the adjusted relative risk of undesirable health status among children, the proxy measure of residual confounding effects, were lifetime abstainer (reference level), 0; former infrequent drinker, 0.11; former regular drinker, −0.12; current infrequent drinker, 0.01; current light drinker, −0.13; current moderate drinker −0.16; current heavier drinker, −0.22; and drinking status unknown, 0.04.

**Figure 1:**
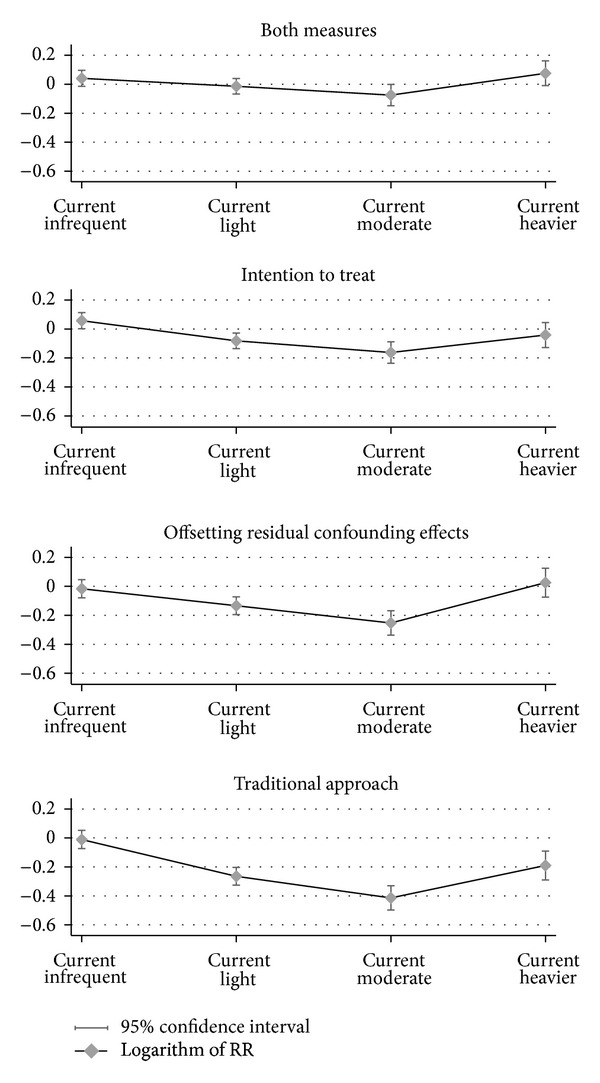
Comparison of alternative models for assessing the association between health status and alcohol consumption: offsetting confounding effects and applying intention-to-treat analysis (RR: relative risk).

Offsetting the residual confounding effects in the model reduced the observed protective effect of light and moderate drinking by 49% and 38% (using logarithmic scale), respectively (Figure 1, Table 1). Returning former drinkers and those with unknown drinking status to their imputed drinker group reduced the observed protective effect of light and moderate drinking by 66% and 60% in logarithmic scale, respectively (Figure 1, Table 1). After combining both adjustments in the same model (i.e., adjustment for confounding effects and applying intention-to-treat analysis), the inverse associations between light drinking and health status and moderate drinking and health status were reduced substantially compared to the results produced using the traditional approach (Figure 1, Tables 1, 3–6).

## 4. Discussion

Using a traditional analytical approach, both light and moderate drinkers appeared to have a reduced likelihood of poor or fair health status when compared to lifetime abstainers. This observation is consistent with findings from many observational studies which have shown apparent protective effects for light and/or moderate drinking for a range of diseases [20–27]. However, making use of such estimates in further analyses, such as those intended to reveal causal relations which may ultimately lead to public health policy or health care advice (e.g., meta-analyses), hinges on the assumption that residual confounding effects are negligible and thereby able to be ignored—even though underlying confounders could be unknown and unmeasured. 

As an alternative, we propose that, rather than assuming residual confounding effects are negligible or in effect, zero, an approximation of the magnitude of residual confounding effects should be obtained by measuring (and adjusting for) the association between alcohol use and the risk of a proxy outcome, whereby the association is mainly manipulated by a similar set of confounding factors. We also argue that former drinkers should be treated as “exposed” subjects, and rather than being analyzed as a separate group, they should be grouped with participants who are currently exposed (i.e., current drinkers) and analysed according to their past drinking status. This is the intention to treat principle used in clinical trials which reduces the potential for bias associated with exposed subjects who have adverse outcomes and subsequently withdraw from treatment. When adjusted for both an estimate of residual confounding and intention to treat, we found that the j-curve for alcohol use and health status which was apparent using the standard approach (unadjusted) was diminished to the point where protective effects were no longer evidenced for low and moderate drinking.

In this example, adult alcohol use was the exposure, and their self-reported health status was the outcome of interest, while children's health status was the proxy outcome measure for gauging residual confounding. Given that it is reasonable to assume that current alcohol use by parents has no direct physiological effect on their children's health, any apparent effects of parental alcohol consumption on their offspring's health could be considered an estimate of true residual confounding effects. It is unlikely that this method will fully account for all bias inherent to observational studies; however, when compared to the current state of play which effectively relegates the magnitude of such bias to zero, it presents an opportunity for advancing a more rational approach. Although children's health status was used as the proxy measure to approximate the magnitude of residual confounding in this study, future observational studies might alternatively measure both the key participant and their spouses' disease outcomes and alcohol use (i.e., where the key participant is a drinker and the cohabiting spouse is a lifetime abstainer). 

In order to apply intention-to-treat analysis it would have been preferable to use recorded previous alcohol use for former drinkers; however, as was the case with the NHIS data, such information may not be available. We therefore explored the option of using multiple imputations to infer former drinkers' past alcohol use status. This method may lead to some degree of misclassification of alcohol use among former drinkers. Nevertheless, it is arguably a more balanced approach than one which removes people who have used alcohol in the past but who stopped drinking due to ill health from the exposure group thereby fostering a nonrandom accumulation of healthier participants into the exposed groups.

The existence of residual confounding effects due to known and unknown confounders in observational studies is highly likely, and that intention-to-treat analysis should be applied, the estimations obtained with the two new adjustments are more close to the true effect comparing to the estimations obtained from the traditional approach. 

## 5. Conclusion

Use of a proxy outcome to estimate and control residual confounding effects of alcohol use and application of the intention-to-treat principle could provide a more realistic estimation of the true effects of alcohol use on health outcomes in observational epidemiological studies. 

## Figures and Tables

**Table 1:** Comparison of alternative models for assessing the association between health status and alcohol consumption: offsetting residual confounding effects and applying intention-to-treat analysis (RR: relative risk).

	RR	95% confidence interval		RR	95% confidence interval		RR	95% confidence interval		RR	95% confidence interval	
Alcohol use	Traditional model			Adjusted for residual confounding effects			Adjusted for intention-to-treat analysis			Adjusted for confounding and intention to treat		
Lifetime abstainer	Reference			Reference			Reference			Reference		
Former infrequent	**1.14**	1.08	1.21	**1.03**	0.97	1.09	∗			∗		
Former regular	**1.21**	1.14	1.28	**1.36**	1.28	1.45	∗			∗		
Current infrequent	**0.99**	0.93	1.05	**0.98**	0.92	1.05	**1.06**	1.00	1.12	**1.04**	0.99	1.10
Current light	**0.77**	0.72	0.82	**0.87**	0.82	0.93	**0.92**	0.87	0.97	**0.99**	0.93	1.04
Current moderate	**0.66**	0.61	0.72	**0.78**	0.71	0.84	**0.85**	0.79	0.92	**0.93**	0.86	1.00
Current heavier	**0.83**	0.75	0.91	**1.03**	0.93	1.13	**0.96**	0.88	1.05	**1.08**	0.99	1.17
Drinking status not reported	**1.04**	0.92	1.18	**1.00**	0.88	1.13	∗			∗		

∗: Intention-to-treat analysis applied.

**Table 2:** Association between alcohol use of key participants and their children's health status, all controlled confounders listed (RR: relative risk).

	RR	95% confidence interval	
Alcohol use of key participant			
Lifetime abstainer	1.00		
Former infrequent	1.11	1.02	1.22
Former regular	0.88	0.78	1.00
Current infrequent	1.01	0.93	1.08
Current light	0.88	0.82	0.94
Current moderate	0.85	0.77	0.95
Current heavier	0.81	0.70	0.93
Drinking status not reported	1.04	0.87	1.24
Age of child			
<1	1.00		
1–5	1.20	1.05	1.38
5–9	1.38	1.21	1.58
10–14	1.56	1.35	1.79
15–17	1.72	1.48	1.99
Gender of child			
Male	1.00		
Female	0.94	0.89	0.99
Gender of key participant			
Male	1.00		
Female	1.07	1.01	1.15
Age group of key participant			
18–24	1.00		
25–34	1.17	1.04	1.32
35–44	1.16	1.03	1.32
45–54	1.11	0.96	1.28
55–64	1.26	1.01	1.56
65–74	1.08	0.72	1.63
75+	1.14	0.46	2.82
Race of key participant			
White	1.00		
Black	1.25	1.17	1.33
Asian	1.24	1.11	1.39
All other race groups	1.29	1.12	1.49
Marital status of key participant			
Married currently living with spouse	1.00		
Married not currently living with spouse	0.96	0.79	1.16
Widowed	1.26	1.02	1.55
Divorced	1.03	0.94	1.13
Separated	1.04	0.94	1.15
Never married	1.11	1.02	1.20
Living with partner	1.08	0.97	1.19
Education level of key participant			
12th grade or lower	1.00		
High-school graduates	0.71	0.67	0.75
Bachelor's degree	0.49	0.44	0.55
Master degree or above	0.44	0.37	0.51
Employment status of key participant			
Working for pay at a job or business	1.00		
With a job or business but not at work	0.98	0.83	1.15
Looking for work	0.97	0.89	1.06
Working, but not for pay, at a family-owned job or business	1.05	0.72	1.51
Not working at a job or business and not looking for work	1.03	0.97	1.10
Total combined family income			
$0–$34,999	1.00		
$35,000–$74,999	0.76	0.70	0.81
$75,000–$99,999	0.61	0.54	0.69
$100,000 and over	0.46	0.41	0.53
Private insurance cover of key participant			
Yes	1.00		
No	1.12	1.05	1.20
Home tenure status of key participant			
Owned or being bought	1.00		
Rented	1.11	1.04	1.18
Other arrangement	0.81	0.68	0.95
Year of survey			
2008	1.00		
2009	0.95	0.89	1.01
2010	1.06	1.00	1.13
Region of residence			
Northeast	1.00		
Midwest	0.94	0.86	1.02
South	0.94	0.86	1.01
West	0.97	0.89	1.05
Number of family members under 18 years of age			
1	1.00		
2	1.03	0.96	1.11
3+	1.11	1.03	1.19
Number of family members aged 65 and older			
0	1.00		
1	1.35	1.11	1.63
2+	0.84	0.45	1.57

**Table 3:** Association between alcohol use and health status using the traditional approach, all controlled confounders listed (RR: relative risk).

	RR	95% confidence interval	
Alcohol use			
Lifetime abstainer	1.00		
Former infrequent	1.14	1.08	1.21
Former regular	1.21	1.14	1.28
Current infrequent	0.99	0.93	1.05
Current light	0.77	0.72	0.82
Current moderate	0.66	0.61	0.72
Current heavier	0.83	0.75	0.91
Drinking status not reported	1.04	0.92	1.18
Gender			
Male	1.00		
Female	0.92	0.89	0.96
Age group			
18–24	1.00		
25–34	2.10	1.82	2.43
35–44	3.45	3.01	3.96
45–54	4.59	4.02	5.24
55–64	5.08	4.44	5.81
65–74	3.24	2.73	3.84
75+	3.38	2.85	4.02
Race			
White	1.00		
Black	1.13	1.08	1.19
Asian	0.91	0.82	1.01
All other race groups	1.26	1.10	1.44
Marital status			
Married currently living with spouse	1.00		
Married not currently living with spouse	0.92	0.79	1.08
Widowed	1.05	0.97	1.12
Divorced	1.12	1.05	1.19
Separated	1.19	1.09	1.29
Never married	1.00	0.94	1.07
Living with partner	1.10	1.00	1.21
Education level			
12th grade or lower	1.00		
High-school graduates	0.79	0.76	0.82
Bachelor's degree	0.54	0.49	0.59
Master degree or above	0.48	0.43	0.54
Employment status			
Working for pay at a job or business	1.00		
With a job or business but not at work	1.51	1.27	1.79
Looking for work	1.41	1.27	1.56
Working, but not for pay, at a family-owned job or business	1.33	1.00	1.78
Not working at a job or business and not looking for work	2.95	2.79	3.12
Total combined family income			
$0–$34,999	1.00		
$35,000–$74,999	0.73	0.69	0.77
$75,000–$99,999	0.54	0.48	0.61
$100,000 and over	0.43	0.38	0.48
Private insurance cover			
Yes	1.00		
No	1.40	1.34	1.47
Home tenure status			
Owned or being bought	1.00		
Rented	1.14	1.09	1.19
Other arrangement	1.16	1.06	1.27
Year of survey			
2008	1.00		
2009	0.97	0.93	1.02
2010	0.96	0.92	1.00
Region of residence			
Northeast	1.00		
Midwest	1.02	0.96	1.08
South	1.13	1.07	1.20
West	1.02	0.95	1.08
Number of family members under 18 years of age			
0	1.00		
1	0.98	0.91	1.04
2	0.92	0.85	1.00
3+	0.81	0.73	0.90
Number of family members aged 65 and older			
0	1.00		
1	0.91	0.83	1.01
2+	1.10	0.97	1.26

**Table 4:** Association between alcohol use and health status using the standard approach plus offsetting for residual confounding effects, all controlled confounders listed (RR: relative risk).

	RR	95% confidence interval	
Alcohol use status			
Lifetime abstainer	1.00		
Former infrequent	1.03	0.97	1.09
Former regular	1.36	1.28	1.45
Current infrequent	0.98	0.92	1.05
Current light	0.87	0.82	0.93
Current moderate	0.78	0.71	0.84
Current heavier	1.03	0.93	1.13
Drinking status not reported	1.00	0.88	1.13
Gender			
Male	1.00		
Female	0.92	0.89	0.96
Age group			
18–24	1.00		
25–34	2.10	1.82	2.43
35–44	3.45	3.01	3.96
45–54	4.59	4.02	5.24
55–64	5.08	4.44	5.81
65–74	3.24	2.73	3.84
75+	3.38	2.85	4.02
Race			
White	1.00		
Black	1.13	1.08	1.19
Asian	0.91	0.82	1.01
All other race groups	1.26	1.10	1.44
Marital status			
Married currently living with spouse	1.00		
Married not currently living with spouse	0.92	0.79	1.08
Widowed	1.05	0.97	1.12
Divorced	1.12	1.05	1.19
Separated	1.19	1.09	1.29
Never married	1.00	0.94	1.07
Living with partner	1.10	1.00	1.21
Education level			
12th grade or lower	1.00		
High-school graduates	0.79	0.76	0.82
Bachelor's degree	0.54	0.49	0.59
Master degree or above	0.48	0.43	0.54
Employment status			
Working for pay at a job or business	1.00		
With a job or business but not at work	1.51	1.27	1.79
Looking for work	1.41	1.27	1.56
Working, but not for pay, at a family-owned job or business	1.33	1.00	1.78
Not working at a job or business and not looking for work	2.95	2.79	3.12
Total combined family income			
$0–$34,999	1.00		
$35,000–$74,999	0.73	0.69	0.77
$75,000–$99,999	0.54	0.48	0.61
$100,000 and over	0.43	0.38	0.48
Private insurance cover			
Yes	1.00		
No	1.40	1.34	1.47
Home tenure status			
Owned or being bought	1.00		
Rented	1.14	1.09	1.19
Other arrangement	1.16	1.06	1.27
Year of survey			
2008	1.00		
2009	0.97	0.93	1.02
2010	0.96	0.92	1.00
Region of residence			
Northeast	1.00		
Midwest	1.02	0.96	1.08
South	1.13	1.07	1.20
West	1.02	0.95	1.08
Number of family members under 18 years of age			
0	1.00		
1	0.98	0.91	1.04
2	0.92	0.85	1.00
3+	0.81	0.73	0.90
Number of family members aged 65 and older			
0	1.00		
1	0.91	0.83	1.01
2+	1.10	0.97	1.26

**Table 5:** Association between alcohol use and health status using the traditional approach plus intention-to-treat analysis, all controlled confounders listed (RR: relative risk).

	IRR	95% confidence interval	
Alcohol use status			
Lifetime abstainer	1.00		
Current infrequent	1.06	1.00	1.12
Current light	0.92	0.87	0.97
Current moderate	0.85	0.79	0.92
Current heavier	0.96	0.88	1.05
Gender			
Male	1.00		
Female	0.93	0.89	0.96
Age group			
18–24	1.00		
25–34	2.12	1.83	2.45
35–44	3.57	3.11	4.10
45–54	4.88	4.28	5.57
55–64	5.47	4.79	6.26
65–74	3.48	2.93	4.13
75+	3.69	3.10	4.39
Race			
White	1.00		
Black	1.14	1.09	1.19
Asian	0.92	0.83	1.02
All other race groups	1.26	1.10	1.45
Marital status			
Married currently living with spouse	1.00		
Married not currently living with spouse	0.91	0.78	1.07
Widowed	1.03	0.96	1.11
Divorced	1.11	1.04	1.17
Separated	1.17	1.07	1.27
Never married	0.99	0.93	1.06
Living with partner	1.08	0.98	1.18
Education level			
12th grade or lower	1.00		
High-school graduates	0.77	0.74	0.81
Bachelor's degree	0.51	0.47	0.56
Master degree or above	0.46	0.41	0.52
Employment status			
Working for pay at a job or business	1.00		
With a job or business but not at work	1.51	1.27	1.79
Looking for work	1.40	1.26	1.55
Working, but not for pay, at a family-owned job or business	1.34	1.00	1.79
Not working at a job or business and not looking for work	3.07	2.90	3.24
Total combined family income			
$0–$34,999	1.00		
$35,000–$74,999	0.71	0.67	0.75
$75,000–$99,999	0.52	0.46	0.58
$100,000 and over	0.40	0.36	0.45
Private insurance cover			
Yes	1.00		
No	1.42	1.36	1.49
Home tenure status			
Owned or being bought	1.00		
Rented	1.15	1.10	1.20
Other arrangement	1.15	1.05	1.26
Year of survey			
2008	1.00		
2009	0.97	0.93	1.02
2010	0.95	0.91	1.00
Region of residence			
Northeast	1.00		
Midwest	1.03	0.97	1.10
South	1.15	1.09	1.22
West	1.02	0.96	1.09
Number of family members under 18 years of age			
0	1.00		
1	0.98	0.92	1.05
2	0.93	0.86	1.01
3+	0.82	0.74	0.91
Number of family members aged 65 and older			
0	1.00		
1	0.92	0.84	1.02
2+	1.10	0.97	1.26

**Table 6:** Association between alcohol use and health status using the traditional approach plus offsetting for residual confounding effects and intention-to-treat analysis (RR: relative risk).

	IRR	95% confidence interval	
Alcohol use status			
Lifetime abstainer	1.00		
Current infrequent	1.04	0.99	1.10
Current light	0.99	0.93	1.04
Current moderate	0.93	0.86	1.00
Current heavier	1.08	0.99	1.17
Gender			
Male	1.00		
Female	0.91	0.88	0.95
Age group			
18–24	1.00		
25–34	2.12	1.83	2.45
35–44	3.53	3.08	4.05
45–54	4.79	4.19	5.46
55–64	5.36	4.69	6.12
65–74	3.42	2.88	4.06
75+	3.61	3.04	4.30
Race			
White	1.00		
Black	1.13	1.08	1.18
Asian	0.91	0.82	1.01
All other race groups	1.27	1.11	1.45
Marital status			
Married currently living with spouse	1.00		
Married not currently living with spouse	0.92	0.78	1.08
Widowed	1.03	0.96	1.11
Divorced	1.12	1.05	1.18
Separated	1.18	1.08	1.29
Never married	1.00	0.93	1.07
Living with partner	1.09	0.99	1.20
Education level			
12th grade or lower	1.00		
High-school graduates	0.78	0.75	0.81
Bachelor's degree	0.52	0.48	0.57
Master degree or above	0.46	0.41	0.52
Employment status			
Working for pay at a job or business	1.00		
With a job or business but not at work	1.51	1.27	1.79
Looking for work	1.40	1.26	1.55
Working, but not for pay, at a family-owned job or business	1.33	0.99	1.79
Not working at a job or business and not looking for work	3.04	2.87	3.21
Total combined family income			
$0–$34,999	1.00		
$35,000–$74,999	0.72	0.68	0.75
$75,000–$99,999	0.53	0.47	0.59
$100,000 and over	0.41	0.37	0.46
Private insurance cover			
Yes	1.00		
No	1.42	1.35	1.49
Home tenure status			
Owned or being bought	1.00		
Rented	1.15	1.10	1.20
Other arrangement	1.16	1.06	1.27
Year of survey			
2008	1.00		
2009	0.97	0.93	1.02
2010	0.96	0.91	1.00
Region of residence			
Northeast	1.00		
Midwest	1.03	0.97	1.09
South	1.15	1.08	1.21
West	1.03	0.96	1.09
Number of family members under 18 years of age			
0	1.00		
1	0.98	0.92	1.05
2	0.93	0.85	1.00
3+	0.82	0.74	0.91
Number of family members aged 65 and older			
0	1.00		
1	0.92	0.83	1.01
2+	1.10	0.96	1.25
